# Drift-Free 3D Orientation and Displacement Estimation for Quasi-Cyclical Movements Using One Inertial Measurement Unit: Application to Running

**DOI:** 10.3390/s22030956

**Published:** 2022-01-26

**Authors:** Marit A. Zandbergen, Jasper Reenalda, Robbert P. van Middelaar, Romano I. Ferla, Jaap H. Buurke, Peter H. Veltink

**Affiliations:** 1 Department of Biomedical Signals and Systems, Faculty of Electrical Engineering, Mathematics and Computer Science (EEMCS), University of Twente, 7522 NB Enschede, The Netherlands; j.reenalda@rrd.nl (J.R.); r.p.vanmiddelaar@utwente.nl (R.P.v.M.); r.i.ferla@student.utwente.nl (R.I.F.); j.buurke@rrd.nl (J.H.B.); p.h.veltink@utwente.nl (P.H.V.); 2 Roessingh Research and Development, 7522 AH Enschede, The Netherlands

**Keywords:** inertial measurement unit, drift-free, orientation, displacement, cyclical, running

## Abstract

A Drift-Free 3D Orientation and Displacement estimation method (DFOD) based on a single inertial measurement unit (IMU) is proposed and validated. Typically, body segment orientation and displacement methods rely on a constant- or zero-velocity point to correct for drift. Therefore, they are not easily applicable to more proximal segments than the foot. DFOD uses an alternative single sensor drift reduction strategy based on the quasi-cyclical nature of many human movements. DFOD assumes that the quasi-cyclical movement occurs in a quasi-2D plane and with an approximately constant cycle average velocity. DFOD is independent of a constant- or zero-velocity point, a biomechanical model, Kalman filtering or a magnetometer. DFOD reduces orientation drift by assuming a cyclical movement, and by defining a functional coordinate system with two functional axes. These axes are based on the mean acceleration and rotation axes over multiple complete gait cycles. Using this drift-free orientation estimate, the displacement of the sensor is computed by again assuming a cyclical movement. Drift in displacement is reduced by subtracting the mean value over five gait cycle from the free acceleration, velocity, and displacement. Estimated 3D sensor orientation and displacement for an IMU on the lower leg were validated with an optical motion capture system (OMCS) in four runners during constant velocity treadmill running. Root mean square errors for sensor orientation differences between DFOD and OMCS were 3.1 ± 0.4° (sagittal plane), 5.3 ± 1.1° (frontal plane), and 5.0 ± 2.1° (transversal plane). Sensor displacement differences had a root mean square error of 1.6 ± 0.2 cm (forward axis), 1.7 ± 0.6 cm (mediolateral axis), and 1.6 ± 0.2 cm (vertical axis). Hence, DFOD is a promising 3D drift-free orientation and displacement estimation method based on a single IMU in quasi-cyclical movements with many advantages over current methods.

## 1. Introduction

Activities like walking, running, swimming, rowing, and skating are all quasi-cyclical in nature. The repetitiveness of these movements, and their associated loads on the human body, can result in overuse injuries [1]. Repetitive movements are often studied inside movement analysis laboratories for insight into overloading of the human body and performance enhancement, among other applications. With the introduction of wearable systems, motion analysis is no longer restricted to a controlled lab setting [2,3]. This opens up new possibilities of analyzing movements that are difficult to measure in a lab, due to technical constraints of optical motion capture systems (OMCS).

Inertial measurement units (IMUs) are widely used in wearable motion capture systems due to their small size and ease of use [4]. IMUs are composed of accelerometers, gyroscopes, and are often combined with magnetometers. The acceleration, orientation, and displacement of a sensor are of interest for many motion analysis applications such as impact analyses, monitoring the range of motion (ROM), or inclination of a body segment, e.g., the lower leg [5,6]. To obtain orientation and displacement from sensor accelerations and angular velocities, the orientation of the sensor in the global coordinate system (CS) ($\Psi^{gl}$) is required. Displacement can then be obtained via strapdown inertial navigation, although this process is prone to errors [7]. Drift in estimated 3D orientations should be minimized as it strongly influences the estimated displacement in $\Psi^{gl}$. Drift can be compensated for by incorporating other sensors (i.e., magnetometer). However, drift reduction and 3D orientation estimation become more challenging during highly dynamic movements, prolonged measurements, or when magnetic distortions are present [8]. Drift can alternatively be reduced by applying domain-specific assumptions, such as the zero-velocity update method [9,10].

The zero-velocity update method assumes that the velocity of the foot is zero and the orientation of the foot is known during the stance phase. This information is used to reset drift in orientation, velocity, and position during each gait cycle [9,10]. Similar assumptions have been used in running in specific conditions. Bailey and Harle corrected for positional drift of an IMU on the foot by using a constant-velocity update in runners with a heel strike [11]. However, constant- or zero-velocity assumptions are not suitable for more proximal segments or runners with a forefoot strike, since a constant- or zero-velocity point is often not present [12].

To estimate orientation and displacement using a single IMU placed on body segments without a constant- or zero-velocity point, the quasi-cyclical nature of numerous movements can be used. Kalman filtering or analytical integration of acceleration in combination with assumptions about the quasi-cyclical nature of movements have been used to estimate displacements of, for example, the pelvis during walking [13,14,15]. These studies involved relatively slow movements, required multiple sensors, a calibration procedure, or prior information about the movements. As a solution to many of these drawbacks, we propose to directly use the quasi-cyclical nature of movements to estimate 3D orientation and displacement using a single IMU without the need for Kalman filtering.

Hence, the research question of this study was: How to estimate 3D orientation and displacement of a single IMU on the lower leg using the quasi-cyclical nature of running?

To answer this research question, a method is proposed in which drift-free 3D orientation and displacement of a single IMU are estimated using the quasi-cyclical nature of numerous human movements. We call this method Drift-Free Orientation and Displacement estimation (DFOD). DFOD assumes that the movement is quasi-cyclical, occurs in a quasi-2D plane, and has an approximately constant cycle average velocity. DFOD will be demonstrated in treadmill running, although it is expected to generalize to many quasi-cyclical quasi-2D movements.

## 2. Materials and Methods

Validation of DFOD was part of a larger study. For sake of clarity, only measurement systems and trials required for validation of DFOD will be described.

### 2.1. Participants

Four healthy recreational runners participated in this study (2M/2F, age: 30.6 ± 9.2 years, height: 181 ± 4 cm, body mass: 65.0 ± 5.4 kg). The study was conducted according to the guidelines of the Declaration of Helsinki, and approved by the Ethics Committee of METC Twente. All participants gave written informed consent before participating in the study.

### 2.2. Protocol

Subjects ran for 2 min on a level treadmill at 3.6 m/s. To validate DFOD with OMCS, a calibration procedure was performed in which subjects stood still in a neutral pose and flexed and extended their leg four times while keeping their upper leg horizontal. This calibration procedure was not required for DFOD but was used to convert the OMCS orientation and displacement estimates to the same CS as used in DFOD for comparison purposes. 

### 2.3. Measurement Systems

**Figure 1:**
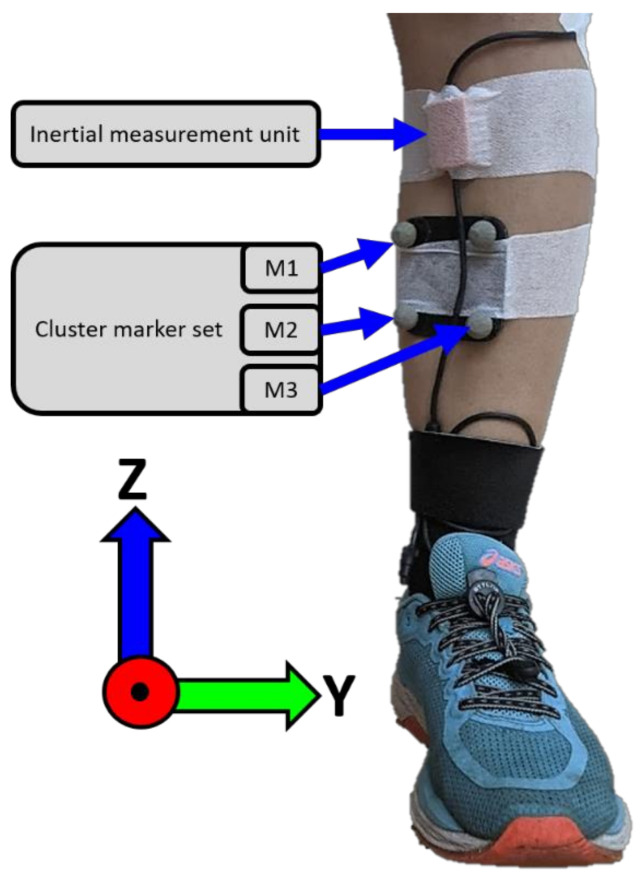
Overview of IMU and cluster marker set placement. “M1”,”M2”, and “M3” refer to the individual markers of the cluster marker set. The shown coordinate system is the functional coordinate system $\Psi^{f}$. The X-axis points forward (running direction), the Y-axis mediolateral, and the Z-axis upward.

Subjects ran on a treadmill (C-Mill, ForceLink, Culemborg, The Netherlands) while 3D angular velocities and accelerations were captured by a single IMU on the lower leg at 240 Hz (MVN Link, Xsens, Enschede, The Netherlands). The ranges of the accelerometer and angular velocity sensor were ±16 g and ±2000°/s, respectively. Positional data from a cluster marker set on the lower leg were captured for reference measurements with an eight-camera optical motion capture system (OMCS) at 100 Hz (Vantage, Vicon, Oxford, UK). The cluster marker set consisted of four individual markers attached to a rigid plate. The IMU was placed medially to the tibial tuberosity and the cluster marker set was placed below the IMU, both on the flat surface of the tibia to ensure measurements of tibia motion, see Figure 1. Both systems were attached to the skin with double-sided tape and covered with stretched strapping tape.

### 2.4. Data Preparation

Optical and inertial data of the left or right lower leg were selected based on minimal OMCS marker occlusion. Optical data were upsampled to 240 Hz with linear interpolation and low-pass filtered with a recursive fourth-order 20 Hz Butterworth filter [16]. Inertial data were not filtered.

**Figure 2:**
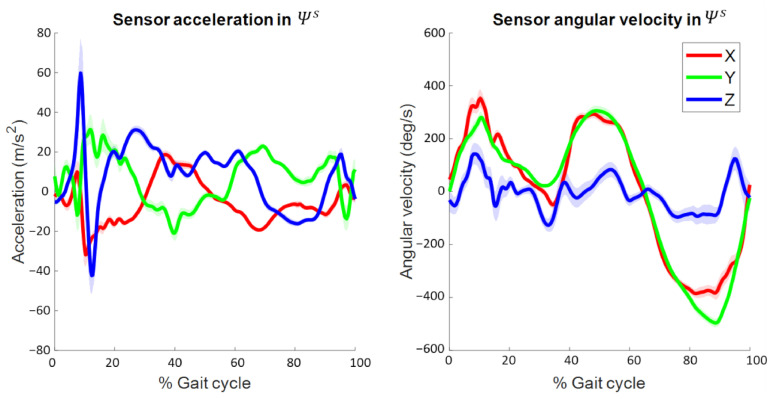
Three-dimensional sensor acceleration (i.e., including gravity) (left figure) and sensor angular velocity (**right**) in $\Psi^{s}$ as a function of the time-normalized gait cycle for a representative subject. Solid lines represent the mean while shaded areas represent the standard deviationaround the mean over one minute of running. Note that these two signals are the input for the orientation and displacement estimation algorithm. Positive acceleration values represent an acceleration into the upward, sideward (**left**), and forward direction of $\Psi^{s}$. Positive angular velocity values represent anti-clockwise rotations in $\Psi^{s}$.

Data were segmented into gait cycles based on falling edge angular velocity zero-crossings in the sensor CS ($\Psi^{s}$) Y-axis, which was directed in the global CS ($\Psi^{gl}$) forward/mediolateral direction. These zero-crossings occur shortly before initial contact. Data were cropped to include all complete gait cycles during one minute of running, hereby excluding around 30–45 s of data in which the subject increased their running speed from standing still up to 3.6 m/s. Sensor acceleration and angular velocity in $\Psi^{s}$ (i.e., input signals for DFOD) as a function of the time-normalized gait cycle for a representative subject are shown in Figure 2. Data analysis was performed in MATLAB R2021a.

### 2.5. Estimate Sensor Orientation in a Functional CS ($\Psi^{f}$)

**Figure 3:**
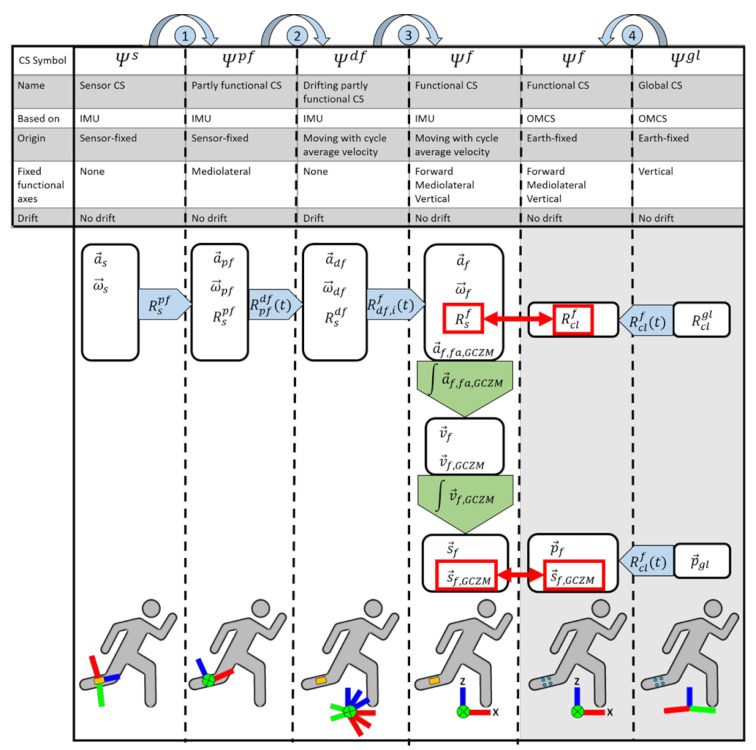
Summary of DFOD (**left four columns**) and the validation of DFOD (**right two columns**). Columns represent different coordinate systems (CS). For each CS some basic information is stated: symbol, name, measurement system on which the CS is based, origin, fixed functional axes (i.e., which axes have functional meaning), and the presence of drift. Available quantities in each CS are shown in white squares (all time-dependent), rotation matrices from one CS to another are shown in blue arrows, curved arrows at the top represent the different rotations which are referred to in the text, integrations over time are shown as green arrows. At the bottom of the figure, a schematic representation of the CS with respect to the lower leg of a runner is shown, orange boxes represent the IMU, and blue dots represent the cluster marker set. DFOD is validated against an OMCS based on the quantities in the red squares. Note that the CSs in grey (two right columns) are only used for validation of DFOD and are not a part of DFOD. IMU = inertial measurement unit; OMCS = optical motion capture system; GCZM = gait cycle zero mean (mean value over each gait cycle is subtracted from the gait cycle); CS = coordinate system; ${\vec{a}}_{CS}\;$= acceleration expressed in the CS in the subscript; ${\vec{\omega }}_{CS}\;$= angular velocity expressed in the CS in the subscript; ${\vec{a}}_{f,fa,GCZM}$ = free acceleration (fa) with a gait cycle zero mean average (GCZM) expressed in the functional CS (f); $R_{CS1}^{CS2}$ = rotation matrix from CS 1 to CS 2; $\vec{v}$ = velocity; $\vec{s}$ = displacement; $\vec{p}$ = position, i = index of gait cycle.

The aim of DFOD is to estimate 3D orientation and displacement of a single sensor in a functional CS ($\Psi^{f}$) of which the vertical and mediolateral axes are fixed and the origin moves with the body at the cycle average velocity. $\Psi^{f}$ is defined in Figure 1; Figure 3. DFOD assumes that the body segment on which the sensor is placed:

- moves quasi-cyclical (i.e., cycles are similar)
- moves in a quasi-2D plane (i.e., most movement occurs in a 2D plane)
- has an approximately constant cycle average velocity

The time- and gait cycle-dependent rotation matrix $R_{s,i}^{f}\left(t\right)$ from $\Psi^{s}$ to $\Psi^{f}$, representing the sensor orientation in a functional drift-free CS of which the vertical and mediolateral axes are fixed and the origin moves with the body at the cycle average velocity, can be written as three subsequent rotations as in Equation (1):

(1) $$R_{s,i}^{f}\left(t\right)=R_{df,i}^{f}R_{pf}^{df}\left(t\right)R_{s}^{pf}$$

where the sensor CS ($\Psi^{s}$) is first rotated to a sensor-fixed partly functional CS ($\Psi^{pf}$) with the time-independent rotation matrix from $\Psi^{s}$ to $\Psi^{pf}$ ($R_{s}^{pf}$). Then, $\Psi^{pf}$ is rotated to a drifting partly functional CS ($\Psi^{df}$) with the time-dependent rotation matrix from $\Psi^{pf}$ to $\Psi^{df}$ ($R_{pf}^{df}\left(t\right)$). $\Psi^{df}$ has an origin that moves with the cycle average velocity. Lastly, drift in $\Psi^{df}$ is corrected for each gait cycle i by rotating to a functional drift free CS ($\Psi^{f}$) with the gait cycle-dependent rotation matrix from $\Psi^{df}$ to $\Psi^{f}$($R_{df,i}^{f}$). All rotations are visualized in Figure 3.

#### 2.5.1. Rotation 1: From Sensor CS ($\Psi^{s}$) to Partly Functional CS ($\Psi^{pf}$)

Integration error accumulation can be reduced by aligning the rotation axis of a quasi-2D movement with one axis in 3D space to create a partly functional CS ($\Psi^{pf}$) [17]. One axis has functional and anatomical meaning in $\Psi^{pf}$. The functional axis (${\vec{y}}_{pf}^{s}$, the y-axis of the sensor-fixed partly functional CS ($\Psi^{pf}$) expressed in the sensor CS ($\Psi^{s}$)) is perpendicular to the plane of movement. Therefore, this axis is described by the first principal component of the angular velocity in $\Psi^{s}$ (${\vec{\omega }}_{s}$), measured by the 3D angular velocity sensor of the IMU, over one minute of running [18]:

(2a) $${\vec{y}}_{pf}^{s}=PCA1\left({\vec{\omega }}_{s}\right)$$

To create a rotation matrix from $\Psi^{pf}$ to $\Psi^{s}$, a temporary X-axis is defined by arbitrarily setting the X-axis of $\Psi^{pf}$ (${\vec{x}}^{,}{}_{pf}^{s}$) to the X-axis of $\Psi^{s}$:

(2b) $${\vec{x}}^{,}{}_{pf}^{s}=\left[1,0,0\right]$$

The Z-axis of $\Psi^{pf}$ (${\vec{z}}_{pf}^{s}$) was computed and ${\vec{x}}^{,}{}_{pf}^{s}$ updated to ensure an orthogonal CS according to the TRIAD algorithm [19]:

(2c) $${\vec{z}}_{pf}^{s}={\vec{x}}^{,}{}_{pf}^{s}\times {\vec{y}}_{pf}^{s}$$

(2d) $${\vec{x}}_{pf}^{s}={\vec{y}}_{pf}^{s}\times {\vec{z}}_{pf}^{s}$$

The time-invariant orthonormal rotation matrix from $\Psi^{pf}$ to $\Psi^{s}$ was:

(2e) $$R_{pf}^{s}=\left[\frac{{\vec{x}}_{pf}^{s}}{\left\|{\vec{x}}_{pf}^{s}\right\|};\frac{{\vec{y}}_{pf}^{s}}{\left\|{\vec{y}}_{pf}^{s}\right\|};\frac{{\vec{z}}_{pf}^{s}}{\left\|{\vec{z}}_{pf}^{s}\right\|}\right]$$

The time-invariant rotation matrix from $\Psi^{s}$ to $\Psi^{pf}$ ($R_{s}^{pf}$) was obtained by taking the inverse of $R_{pf}^{s}$ Equation (2e):

(2f) $$R_{s}^{pf}=R_{pf}^{s}{}^{-1}$$

#### 2.5.2. Rotation 2: From Partly Functional CS ($\Psi^{pf}$) to Drifting Partly Functional CS ($\Psi^{df}$)

To go from a sensor-fixed CS to a drifting CS in which axes do not depend on the sensor orientation, the angular velocity in $\Psi^{pf}$(${\vec{\omega }}_{pf}$) was integrated according to Bortz [20,21]. ${\vec{\omega }}_{pf}$ was expressed as a skew-symmetric matrix (${\tilde{\omega }}_{pf}$), and the differential equation was solved and used to obtain the rotation matrix from $\Psi^{pf}$ to $\Psi^{df}$ ($R_{pf}^{df}$):

(3a) $${\tilde{\omega }}_{pf}\left(t\right)=\left(\begin{array}{ccc} 0 & -\omega_{pf,z}\left(t\right) & \omega_{pf,y}\left(t\right)\\ \omega_{pf,z}\left(t\right) & 0 & -\omega_{pf,x}\left(t\right)\\ -\omega_{pf,y}\left(t\right) & -\omega_{pf,x}\left(t\right) & 0 \end{array}\right)\;$$

(3b) $${\dot{R}}_{pf}^{df}\left(t\right)=R_{pf}^{df}\left(t\right){\tilde{\omega }}_{pf}\left(t\right)$$

${\dot{R}}_{pf}^{df}\left(t\right)\;$ is the time-derivative of $R_{pf}^{df}\left(t\right)$, and $R_{pf}^{df}\left(t\right)$ at *t* = 0 is the identity matrix. Note that $\Psi^{df}$ drifts, predominantly around the *y*-axis ($\Psi_{y}^{df}$), due to accumulated integration errors from Equation (3a,b). This drift needs to be corrected to get a useful orientation estimate of the sensor (rotation 3 in Figure 3).

#### 2.5.3. Rotation 3: From Drifting Partly Functional CS ($\Psi^{df}$) to Drift-Free Functional CS ($\Psi^{f}$)

Following an assumption of quasi-cyclical running, the lower leg keeps rotating around the same mediolateral axis. By continuously calculating this rotation axis we can correct for integration drift from Equation (3a,b). The rotation axis was again based on the first principal component of the 3D angular velocity, now in $\Psi^{df}$ (${\vec{\omega }}_{df})$, over five complete gait cycles Equation (4a). Multiple gait cycles were included to obtain a more robust estimate of ${\vec{y}}_{df,i}^{f}$ (see also Section 2.7 for algorithm characteristics):

(4a) $${\vec{y}}_{df,i}^{f}=PCA1({\vec{\omega }}_{df}\left(t\right))\;\;t_{0_{i}}-T_{i-1}-T_{i-2}<t<t_{0_{i}}+T_{i}+T_{i+1}+T_{i+2}$$

where $t_{0_{i}}-T_{i-1}-T_{i-2}<t<t_{0_{i}}+T_{i}+T_{i+1}+T_{i+2}$ represents the interval of five complete gait cycles, $t_{0_{i}}$ stands for the first time point of gait cycle i, $T_{i}\;$ stands for the duration of gait cycle i, and is obtained from the earlier described falling edge angular velocity zero-crossings in $\Psi_{y}^{s}$ (Section 2.4). Note that the first principal component of the angular velocity is obtained twice Equations (2a) and (4a). In Equation (2a), ${\vec{y}}_{s}^{pf}$ has a constant value over a longer period of time since there is no drift in $\Psi^{s}$. In Equation (4a), the angular velocity ${\vec{\omega }}_{df}$ is expressed in a drifting CS ($\Psi^{df}$). Therefore, ${\vec{y}}_{df,i}^{f}$ differs for each gait cycle to correct for the drift in $\Psi^{df}$.

Following an assumption of approximately constant cycle average velocity running, the free acceleration in $\Psi^{f}$ will be approximately zero-mean over a complete gait cycle. Hence, the mean total acceleration (i.e., including gravity) over a complete number of gait cycles represents the gravitational acceleration and is directed vertically. Therefore, the temporary Z-axis of the functional CS $\Psi^{f}$ (${\vec{z}}^{,}{}_{df}^{f}$) was based on the average total acceleration (i.e., including gravity) in $\Psi^{df}$ (${\vec{a}}_{df}$), over five complete gait cycles Equation (4b). Multiple gait cycles were included to obtain a more robust estimate of ${\vec{z}}^{,}{}_{df}^{f}$ (see also Section 2.7 for algorithm characteristics):

(4b) $${\vec{z}}^{,}{}_{df,i}^{f}=\frac{1}{\sum_{j=-2}^{+2}T_{i+j}}\int_{t_{0_{i}}-T_{i-1}-T_{i-2}}^{t_{0_{i}}+T_{i}+T_{i+1}+T_{i+2}}{\vec{a}}_{df}\left(\tau \right)d\tau$$

where j is an index to define included gait cycles. The *x*-axis of $\Psi^{df}$ (${\vec{x}}_{df}^{f}$) was computed and ${\vec{z}}^{,}{}_{df,i}^{f}$ updated to ensure an orthogonal CS according to the TRIAD algorithm [19]:

(4c) $${\vec{x}}_{df,i}^{f}={\vec{y}}_{df,i}^{f}\times {\vec{z}}^{,}{}_{df,i}^{f}$$

(4d) $${\vec{z}}_{df,i}^{f}={\vec{x}}_{df,i}^{f}\times {\vec{y}}_{df,i}^{f}$$

The orthonormal drift-correcting rotation matrix from $\Psi^{df}$ to $\Psi^{f}$ was:

(4e) $$R_{df,i}^{f}=\left[\frac{{\vec{x}}_{df,i}^{f}}{\left\|{\vec{x}}_{df,i}^{f}\right\|};\frac{{\vec{y}}_{df,i}^{f}}{\left\|{\vec{y}}_{df,i}^{f}\right\|};\frac{{\vec{z}}_{df,i}^{f}}{\left\|{\vec{z}}_{df,i}^{f}\right\|}\right]$$

where $R_{df,i}^{f}$ has a constant value within each cycle but varies over cycles to correct for drift. The drift-free 3D rotation matrix of the sensor in a functional CS ($R_{s,i}^{f}$) of which the vertical and mediolateral axes are fixed, and the origin moves with the body at the cycle average velocity was then computed with Equation (1).

#### 2.5.4. From Sensor Orientation to Sensor Displacement

Three-dimensional angular velocity and total (i.e., including gravity) acceleration in $\Psi^{f}$ (${\vec{\omega }}_{f}\;\mathrm{and}\;{\vec{a}}_{f}$) were obtained with Equation (1). Free acceleration in $\Psi^{f}$ (${\vec{a}}_{f,fa})\;$ was obtained by subtracting the modulus of the gravitational acceleration (${\vec{g}}_{f}$) from the total acceleration (${\vec{a}}_{f}$):

(5a) $${\vec{a}}_{f,fa}\left(t\right)={\vec{a}}_{f}\left(t\right)-\left[0,\;0,\;\|{\vec{g}}_{f}\|\right]$$

Following an assumption of approximately constant cycle average velocity, the free acceleration in $\Psi^{f}$ will be approximately zero-mean over a complete number of gait cycles. Hence, the mean free acceleration value over a window of five gait cycle was subtracted to correct for drift:

(5b) $${\vec{a}}_{f,fa,GZCM}\left(t\right)={\vec{a}}_{f,fa}\left(t\right)-\frac{1}{T_{i}}\int_{t_{0_{i}}-T_{i-1}-T_{i-2}}^{t_{0_{i}}+T_{i}+T_{i+1}+T_{i+2}}{\vec{a}}_{f,fa}\left(\tau \right)d\tau \;\;t_{0_{i}}\leq t\;and\;t_{0_{i}}+T_{i}>t\;$$

where ${\vec{a}}_{f,fa,GZCM}$ is the free acceleration with a gait cycle zero-mean (GCZM). ${\vec{a}}_{f,fa,GZCM}$ was numerically integrated (Figure 3, Column $\Psi^{f}$, upper green arrow) with the trapezoidal rule to obtain the velocity (${\vec{v}}_{f}$):

(6a) $${\vec{v}}_{f}\left(t\right)=\int_{t_{0_{i}}}^{t}{\vec{a}}_{f,fa,GZCM}\left(\tau \right)d\tau$$

Following an assumption of approximately constant cycle average velocity, the mean velocity in $\Psi^{f}$ over a complete number of gait cycles is approximately zero in all axes since the origin of $\Psi^{f}$ moves with the body at the cycle average velocity. Hence, the drift-corrected GCZM velocity was computed by subtracting the mean velocity over a window of five gait cycles:

(6b) $${\vec{v}}_{f,GCZM}\left(t\right)={\vec{v}}_{f}\left(t\right)-\frac{1}{T_{i}}\int_{t_{0_{i}}-T_{i-1}-T_{i-2}}^{t_{0_{i}}+T_{i}+T_{i+1}+T_{i+2}}{\vec{v}}_{f}\left(\tau \right)d\tau \;\;\;t_{0_{i}}\leq t\;and\;t_{0_{i}}+T_{i}>t$$

Sensor displacement in $\Psi^{f}$ (${\vec{s}}_{f}$) was obtained with the trapezoidal rule and numeric integration of ${\vec{v}}_{f,GCZM}$ (Figure 3, Column $\Psi^{f}$, lower green arrow):

(7a) $${\vec{s}}_{f}\left(t\right)=\int_{t_{0_{i}}}^{t}{\vec{v}}_{f,GCZM}\left(\tau \right)d\tau$$

Following an assumption of approximately constant cycle average velocity, the mean displacement in $\Psi^{f}$ approximates zero is all directions over a complete number of gait cycles. Hence, the mean displacement over five gait cycles was subtracted and GCZM displacement (${\vec{s}}_{f,GCZM}$) was computed and used as outcome measure:

(7b) $${\vec{s}}_{f,GCZM}\left(t\right)={\vec{s}}_{f}\left(t\right)-\frac{1}{T_{i}}\int_{t_{0_{i}}-T_{i-1}-T_{i-2}}^{t_{0_{i}}+T_{i}+T_{i+1}+T_{i+2}}{\vec{s}}_{f}\left(\tau \right)d\tau \;\;t_{0_{i}}\leq t\;and\;t_{0_{i}}+T_{i}>t$$

### 2.6. Validation of Orientation and Displacement Estimates

The steps described below are used to validate DFOD and are not part of DFOD. To compare the results of DFOD against OMCS, the orientation of the cluster marker set was computed and both the orientation and displacement were transformed to $\Psi^{f}$.

#### 2.6.1. Rotation 4: From Optical Motion Capture CS ($\Psi^{cl})$ to Functional CS ($\Psi^{f}$)

The orientation of the OMCS cluster marker set in $\Psi^{gl}\;$ was based on the relative positions of three of its individual markers according to the TRIAD algorithm [19]:

(8a) $${\vec{z}}_{cl}^{gl}\left(t\right)={\vec{p}}_{gl,m2}\left(t\right)-{\vec{p}}_{gl,m1}\left(t\right)$$

(8b) $${\vec{y}}^{,}{}_{cl}^{gl}\left(t\right)={\vec{p}}_{gl,m3}\left(t\right)-{\vec{p}}_{gl,m1}\left(t\right)$$

where ${\vec{p}}_{gl,m}$ refers to the position of the individual markers of the cluster marker set in $\Psi^{gl}$, see Figure 1. ${\vec{z}}_{cl}^{gl}$ and ${\vec{y}}^{,}{}_{cl}^{gl}$ represent the *Z*-axis and temporary Y-axis of the cluster marker CS ($\Psi^{cl})$ in $\Psi^{gl}$. The X-axis of $\Psi^{cl}$ (${\vec{x}}_{cl}^{gl}$) was computed and ${\vec{y}}^{,}{}_{cl}^{gl}$ updated to ensure an orthogonal CS:

(8c) $${\vec{x}}_{cl}^{gl}\left(t\right)={\vec{y}}^{,}{}_{cl}^{gl}\left(t\right)\times {\vec{z}}_{cl}^{gl}\left(t\right)$$

(8d) $${\vec{y}}_{cl}^{gl}\left(t\right)={\vec{z}}_{cl}^{gl}\left(t\right)\times {\vec{x}}_{cl}^{gl}\left(t\right)$$

The orthonormal rotation matrix of $\Psi^{cl}$ to $\Psi^{gl}$ ($R_{cl}^{gl}$) was:

(8e) $$R_{cl}^{gl}\left(t\right)=\left[\frac{{\vec{x}}_{cl}^{gl}\left(t\right)}{\left\|{\vec{x}}_{cl}^{gl}\left(t\right)\right\|};\frac{{\vec{y}}_{cl}^{gl}\left(t\right)}{\left\|{\vec{y}}_{cl}^{gl}\left(t\right)\right\|};\frac{{\vec{z}}_{cl}^{gl}\left(t\right)}{\left\|{\vec{z}}_{cl}^{gl}\left(t\right)\right\|}\right]$$

To be comparable, the 3D orientation and position of the OMCS cluster marker set need to be expressed in the same functional CS as used for the sensor orientation and displacement estimate of DFOD. Therefore, the OMCS functional Y-axis ($\Psi_{y}^{f}$) in$\;\Psi^{gl}$ (${\vec{y}}_{f}^{gl}$) should be based on the same functional axis as in Equation (4a). However, differentiating $R_{cl}^{gl}\left(t\right)$ and computing the first principal component is prone to stochastic errors induced by differentiating 3D orientations. Alternatively, we estimated the rotation axis of the lower leg (${\vec{y}}_{f}^{gl}$) during the flexion–extension movements of the calibration trial, described in Section 2.2. During the flexion–extension movements, the lower leg moves approximately around the same rotation axis. This rotation axis (${\vec{y}}_{f}^{gl}$) was estimated by first dividing each of the four flexion–extension movements into seven intervals of equal duration ($\frac{T_{i}}{7}$), $T_{i}$ being the duration of cycle i. See Section 2.7 for algorithm characteristics. By using a larger time interval, the change in rotation during this interval is relatively large compared to the errors. The rotation matrix from time point $t_{j}$=$\;t_{i}+j\times \frac{T_{i}}{7}$ to the next was then computed as follows:

(9a) $$R_{t_{j}}^{t_{j+1}}=R_{cl}^{gl}{\left(t_{j}\right)}^{-1}R_{cl}^{gl}\left(t_{j+1}\right)$$

Subsequently, $R_{t_{j}}^{t_{j+1}}$ of cycle i was transformed to a rotation axis (${\vec{v}}_{rot,i,j}$) which corresponds to the vector part of a quaternion that can be derived from a rotation matrix [22]. ${\vec{v}}_{rot,i,j}\;$ was multiplied by a factor −1 for the extension part of each calibration movement cycle to ensure that the rotation axes were approximately equally directed for all intervals. The functional coordinate axis ${\vec{y}}_{f}^{gl}\;$(i.e., the rotation axis of the lower leg during the flexion–extension movements) was subsequently determined by averaging all resulting rectified rotation axes ${\vec{v}}_{rot,i,j}^{,}$ for all intervals j and all cycles i:

(9b) $${\vec{y}}_{f}^{gl}=\frac{1}{4\times 7}\sum_{i=1}^{4}\sum_{j=1}^{7}{\vec{v}}_{rot,i,j}^{,}$$

The temporary *Z*-axis of $\Psi^{f}\;$(${\vec{z}}^{,}{}_{f}^{gl}$) was chosen to be equal to $\Psi_{z}^{gl}$:

(9c) $${\vec{z}}^{,}{}_{f}^{gl}=\left[0,0,1\right]$$

The X-axis of $\Psi^{f}$ (${\vec{x}}_{f}^{gl}$) was computed and ${\vec{z}}^{,}{}_{f}^{gl}$ corrected to create an orthogonal CS:

(9d) $${\vec{x}}_{f}^{gl}={\vec{y}}_{f}^{gl}\times {\vec{z}}^{,}{}_{f}^{gl}$$

(9e) $${\vec{z}}_{f}^{gl}={\vec{x}}_{f}^{gl}\times {\vec{y}}_{f}^{gl}$$

The orthonormal time-invariant rotation matrix from $\Psi^{f}$ to $\Psi^{gl}$ ($R_{f}^{gl}$) was:

(9f) $$R_{f}^{gl}=\left[\frac{{\vec{x}}_{f}^{gl}}{\left\|{\vec{x}}_{f}^{gl}\right\|};\frac{{\vec{y}}_{f}^{gl}}{\left\|{\vec{y}}_{f}^{gl}\right\|};\frac{{\vec{z}}_{f}^{gl}}{\left\|{\vec{z}}_{f}^{gl}\right\|}\right]$$

The time-dependent rotation matrix of $\Psi^{cl}$ in $\Psi^{f}$ ($R_{cl}^{f}$) was then computed and represented the orientation of the cluster marker set in $\Psi^{f}$ (rotation 4 in Figure 3):

(10) $$R_{cl}^{f}\left(t\right)=R_{f}^{gl}{}^{-1}R_{cl}^{gl}\left(t\right)$$

#### 2.6.2. Orientation and Displacement Validation

The sensor and cluster orientation estimates in $\Psi^{f}$ of DFOD and OMCS were expressed in Euler angles (rotation order: YZX) for visualization purposes. To show the added drift-reducing benefit of DFOD in estimating sensor orientation, sensor orientation was also computed by integrating the sensor angular velocity in $\Psi^{s}$, similar to Equation (3a,b) without any drift reducing methods. This resulted in the sensor orientation with respect to the initial sensor orientation ($\Psi^{s,init}$) at the start of the first gait cycle. The position of the marker closest to the IMU was selected and displacement during each gait cycle was computed (similar procedure to Equation (7b)). OMCS and IMU data were time-synchronized based on the GCZM displacement in the forward direction of the sensor and cluster marker set (${\vec{s}}_{f,GCZM,x}$). Three-dimensional differences in Euler angles and displacement between DFOD and OMCS over time-normalized gait cycles were quantified as root mean square errors (RMSE) and absolute mean differences. A 1D orientation error was computed by transforming the difference in orientation between DFOD and OMCS to an axis-angle representation and using the rotation angle as an outcome [23]. This 1D angle represents the rotation that is necessary to align $R_{s}^{f}$ and $R_{cl}^{f}$. A 1D displacement error was defined as the root mean square of the 3D displacement errors. Additionally, differences at the first and last sample of each gait cycle, differences in minimum and maximum values, and the ROM between DFOD and OMCS for each gait cycle were computed and correlations between extrema and ROM were quantified with Pearson correlation coefficients. Correlations are interpreted as very strong for r = (0.90, 1.00), strong for r = (0.70, 0.89), moderate for r = (0.40, 0.69), weak for r = (0.20, 0.39), and very weak for r = (0.00, 0.19) [24]. The mediolateral and vertical axis of DFOD Equation (4a,b) were based on five gait cycles unless stated otherwise.

### 2.7. Algorithm Characteristics

DFOD assumes that the sensor on the lower leg moves quasi-cyclically and in a quasi-2D plane. To quantify how valid these assumptions are for the lower leg motion during treadmill running, respectively the mean cycle time and standard deviation and the explained variance of the first principal component of the angular velocity in $\Psi^{s}$ over one minute of running were computed.

The mediolateral (Equation (4a), ${\vec{y}}_{df,i}^{f}$) and vertical axes (Equation (4b), ${\vec{z}}_{df,i}^{f}$) of $\Psi^{f}$ can be computed independently of each other and are not necessarily based on data of the same number of gait cycles. The effect of using data of different numbers of gait cycles to determine these axes of $\Psi^{f}$ and its error with respect to an OMCS was tested. Data of 1 up to 15 gait cycles were used to define the mediolateral and vertical axes of $\Psi^{f}$, resulting in a total of 225 combinations which were tested. The outcome measure of this analysis was the 1D orientation and displacement estimate.

Full trust in the TRIAD algorithm [19] was given to the mediolateral functional axis Equation (4a) since this axis is not influenced by the violation of the approximately constant cycle average velocity assumption. The number of points used to estimate the rotation axis of Equation (9a,b) was based on a trial and error procedure to obtain a small variation in the obtained axes while using as few intervals as possible. Note that the results of this trial and error process were only used to validate DFOD and were not part of DFOD.

To investigate the effect of sampling frequency on the performance of DFOD, IMU data were resampled from 240 Hz to 120 Hz and 60 Hz before DFOD was used to estimate orientation and displacement. For this analysis, the vertical and mediolateral axis of DFOD were both based on five gait cycles and the 1D orientation and displacement estimates were used as outcome measures.

## 3. Results

An average of 79 gait cycles (range: 66–94) per subject were analyzed. When not stated otherwise, the mediolateral and vertical axes of DFOD Equation (4a,b) were based on data of five gait cycles.

### 3.1. Estimation of Orientation

**Figure 4:**
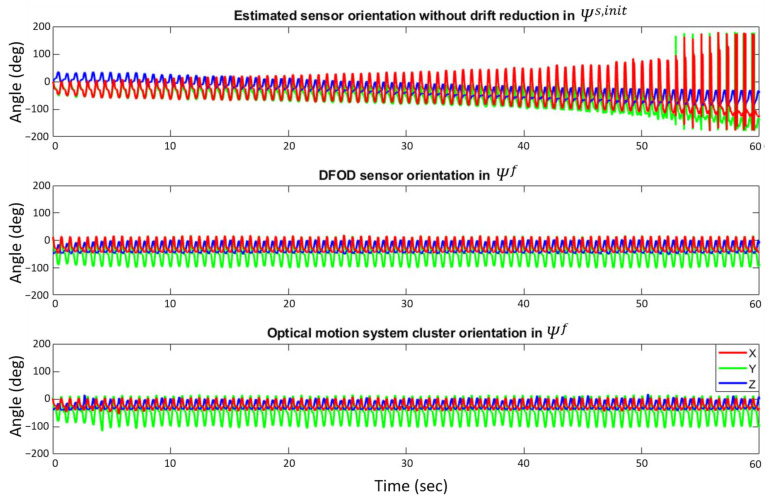
Estimated sensor (inertial) and cluster marker set (optical) orientation for a representative subject. The top figure shows estimated sensor orientation without drift reduction with respect to the initial orientation of the sensor at the start of the first gait cycle ($\Psi^{s,init}$). The middle figure shows the estimated sensor orientation obtained with DFOD in $\Psi^{f}$. The bottom figure shows the actual cluster orientation according to an optical motion capture system in $\Psi^{f}$. Note that data of the top graph are shown in a different coordinate system. This figure shows the added drift-reducing benefit of DFOD compared to orientation estimation without drift reduction. Anti-clockwise rotations in $\Psi^{s,init},$ (top figure), and $\Psi^{f}$ (middle and bottom figure) correspond to positive angles. An angle of zero corresponds to the initial sensor orientation just before initial contact of the first gait cycle in $\Psi^{s,init}$ (top figure) or $\Psi^{f}$ (middle and bottom figure).

Estimated lower leg sensor orientations without drift reduction, with drift reduction according to DFOD and from an OMCS are shown in Figure 4.

**Figure 5:**
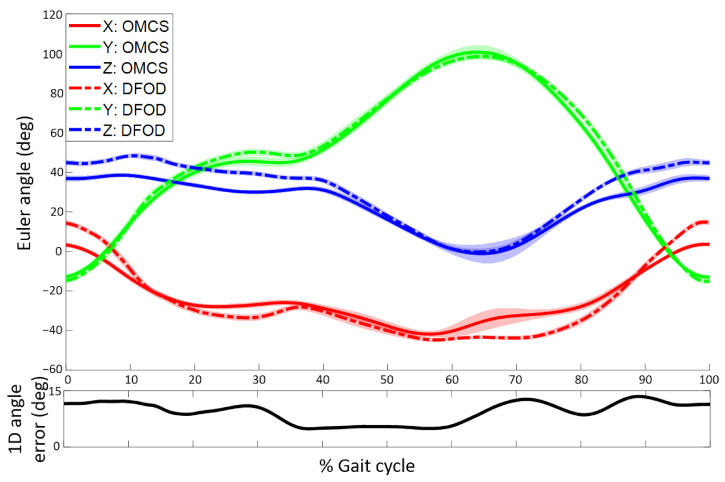
Top figure: Mean time-normalized orientation of a sensor (DFOD, dashed line) and cluster marker (OMCS, solid line) on the lower leg (in $\Psi^{f}$) as a function of the gait cycle. Shaded areas represent the standard deviation around the mean. Bottom figure: 1D orientation error as a function of the gait cycle. The 1D orientation error is the angle of the axis-angle representation of the difference in orientation between DFOD and OMCS [23]. Data are shown for a representative subject during one minute of running. Positive orientations represent anti-clockwise rotations in $\Psi^{f}$.

Estimated lower leg sensor orientations of DFOD were compared to an OMCS in treadmill running. Mean RMSE for orientations in the sagittal plane were 3.1 ± 0.4° while they were larger in the frontal (5.3 ± 1.1°) and transversal plane (5.0 ± 2.1°). The mean 1D rotation error (i.e., angle over which $R_{s}^{f}$ needs to be rotated to coincide with $R_{cl}^{f}$) was 7.5 ± 1.7°. The 3D mean difference at the start and end of the gait cycle, absolute difference, and maximum and minimum difference in orientation together with the difference in ROM of DFOD and OMCS are shown in Table 1. Correlations between the 3D maximal angle, minimal angle, and ROM from DFOD and OMCS ranged from strong (0.768) to very strong (0.99). Mean 3D orientations of DFOD and OMCS for a representative subject are shown in Figure 5.

### 3.2. Estimation of Displacement

**Figure 6:**
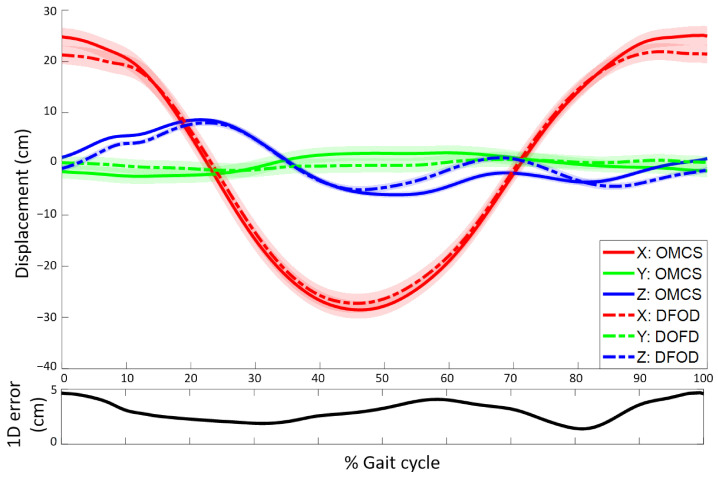
(**Top** figure): Mean time-normalized displacement of a sensor (DFOD, dashed line) and cluster marker (OMCS, solid line) on the lower leg (in $\Psi^{f}$) as a function of the gait cycle. Shaded areas represent the standard deviation around the mean. (**Bottom** figure): 1D displacement error as a function of the gait cycle. Data are shown for a representative subject during one minute of running. Positive displacements in the X, Y, and Z-axis represent movement into the forward, sideward (left), and upward direction, respectively. The origin moves forward with the cycle average velocity.

Estimated lower leg sensor displacements of DFOD were compared to an OMCS in treadmill running. Mean RMSE for displacements in the forward direction were 1.6 ± 0.2 cm and similar for the mediolateral (1.7 ± 0.6 cm) and vertical direction (1.6 ± 0.2 cm). The mean 1D displacement error (i.e., length of the vector between the estimated sensor position of DFOD and OMCS) was 2.7 ± 0.4 cm. The 3D mean difference at the start and end of the gait cycle, absolute difference, maximum difference, and minimum difference in displacement, together with the difference in ROM of DFOD and OMCS, are shown in Table 2. Correlations between the 3D maximal displacement, minimal displacement, and ROM were moderate (r = 0.50) to strong (r = 0.82). Mean 3D displacements of DFOD and OMCS for a representative subject are shown in Figure 6.

### 3.3. Algorithm Characteristics

Two metrics were computed to show how valid the assumptions of a quasi-cyclical and quasi-2D movements were for treadmill running. The average cycle time was 0.68 ± 0.03 s/stride and the standard deviation ranged from 0.8–1.9% of the average cycle time. The first principal component of the angular velocity explained on average 90.2 ± 5.7% (range: 84.6–95.8%) of the variance.

**Figure 7:**
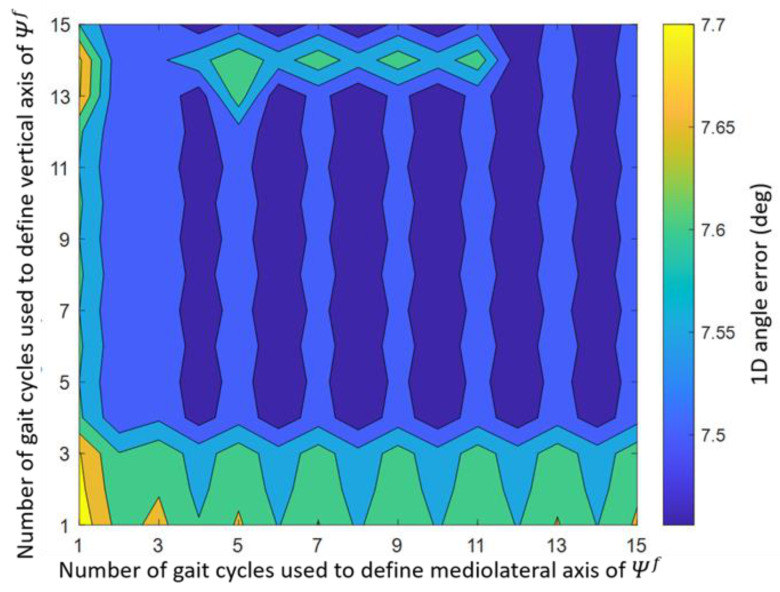
Effect of the number of gait cycles used to determine the mediolateral Equation (4a) and vertical axis Equation (4b) of $\Psi^{f}$ on the 1D angle error. The 1D angle error represents the angle of the axis-angle representation of the difference in orientation between DFOD and OMCS.

The mediolateral and vertical axes of $\Psi^{f}$ Equation (4a,b) are based on data of five complete gait cycles. The effect of using data of more or less gait cycles to define these axes on the mean 1D orientation error is investigated and shown in Figure 7. The lowest mean 1D orientation error was found when the vertical axis was based on data of 11 gait cycles and the mediolateral axis on data of 8 gait cycles (mean error: 7.5°). The highest mean orientation error was found when the vertical and mediolateral axes were both based on data of 1 gait cycle (mean error: 7.7°).

**Figure 8:**
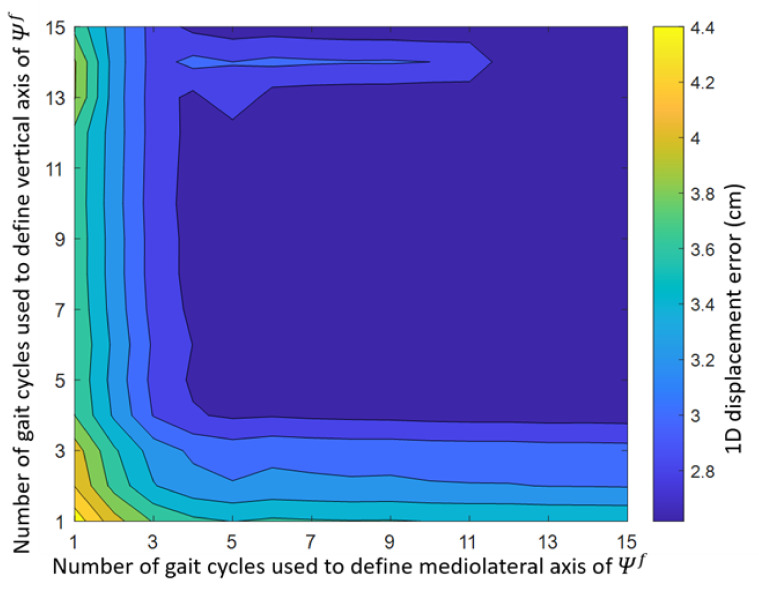
Effect of the number of gait cycles used to determine the mediolateral Equation (4a) and vertical axis Equation (4b) of $\Psi^{f}$ on the 1D displacement error between DFOD and OMCS.

The effect of the number of gait cycles on the mean 1D displacement error is shown in Figure 8. The lowest mean displacement error was found when the vertical axis was based on data of 10 gait cycles and the mediolateral axis on data of 15 gait cycles (mean error: 2.6 cm). The highest mean displacement error was found when the vertical and mediolateral axes were both based on data of 1 gait cycle (mean error: 4.5 cm).

To investigate the effect of sampling frequencies on DFOD, inertial data were resampled from 240 Hz to 120 Hz and 60 Hz before applying DFOD. Compared to a sampling frequency of 240 Hz, the 1D orientation error increased with 0.3° for 120 Hz and 2.2° for 60 Hz. The 1D displacement error increased with 1.2 cm for 120 Hz and 12.7 cm for 60 Hz.

## 4. Discussion

A new method, called Drift-Free Orientation and Displacement estimation (DFOD), is proposed to estimate drift-free 3D sensor orientation and displacement based on a single IMU. DFOD uses the quasi-cyclical behavior of human movements and assumes a quasi-2D movement with an approximately constant cycle average velocity. The performance of DFOD for a sensor on the lower leg was validated with an optical motion capture system (OMCS) in treadmill running. Errors in estimated sensor orientation and displacement between DFOD and OMCS were comparable to errors of other orientation and displacement algorithms. However, DFOD is independent of a constant- or zero-velocity point, a biomechanical model, a magnetometer, Kalman filtering, or a calibration procedure. Hence, DFOD is a promising method for quasi-cyclical motion analysis with a single IMU and has many advantages over current methods.

### 4.1. Estimation of Orientation

Estimated lower leg sensor orientations of DFOD were compared to an OMCS in treadmill running. DFOD performs best for orientation estimation in the sagittal plane, possibly because the largest ROM occurs around the axis perpendicular to this plane Equation (4a) in running.

To reduce drift in orientation estimation, a drift reducing rotation which was constant within each cycle, but varied over cycles, was applied (rotation 3). Orientation drift is relatively slow compared to the duration of a gait cycle (i.e., two min before $\Psi^{df}$ drifts 90°, or ±0.5°/stride, around $\Psi_{y}^{f}$). Hence, a constant drift reducing rotation for each gait cycle seemed sufficient, although this did result in small discontinuities between gait cycles. In future work, a continuous drift reducing rotation could improve the performance of DFOD.

Since we are not aware of studies that estimated lower leg orientations during running, the results of DFOD can only be compared with studies estimating foot and thigh orientations during running and walking. Foot orientations during running have mostly been based on constant- or zero-velocity updates with an additional drift reducing component (e.g., based on joint center accelerations, filtering, or an orientation reset). At speeds similar to our study, sagittal plane foot orientations could be estimated with errors varying between 2.0° and 20.8° [11,12,25]. Frontal plane foot orientation errors differed from 2.6° to 4.4° [12,25]. Upper leg orientations during walking have been estimated with an RMSE of 1.9 ± 0.5°, although the zero acceleration and angular velocity update used in that study does not apply to continuous quasi-cyclical movements like running [26]. Orientation errors in our study are similar or slightly larger than found in literature for other body segments, although these studies used drift reducing methods unsuitable for a sensor on the lower leg in running (i.e., based on a constant- or zero-velocity point).

Tibial orientations in the sagittal and transversal plane are commonly studied with regard to running injuries [27,28,29]. The sagittal plane orientation of the tibia at initial contact has been shown to be 4.9° larger in injured than in uninjured runners and the tibia ROM in the transverse plane is around 15° in running [30]. With a mean difference of –0.3 ± 3.7° at the start of the gait cycle (just before initial contact) and –5.6 ± 2.1° in the transversal plane ROM, DFOD is capable to detect meaningful changes in tibia orientations during running.

### 4.2. Estimation of Displacement

Estimated lower leg sensor displacements of DFOD were compared to an OMCS in treadmill running. OMCS cluster marker placement can explain some of the errors in the forward and vertical directions. The OMCS cluster marker set is placed below the IMU (see Figure 1). Lower placement of the cluster marker set results in a larger ROM for OMCS compared to DFOD in the forward and vertical direction. Hence, actual displacement errors in the forward and vertical direction are expected to be smaller than those reported in this study.

Since we are not aware of studies that estimated lower leg displacements during running, the results of DFOD can only be compared with studies estimating foot displacements and stride length based on IMU data during running. In literature, estimates of sagittal plane foot displacement during running at a speed similar to the speed in this study had an absolute 1D positional error of 5 ± 2 cm at maximal foot height and initial contact [11]. The absolute 1D positional error in our study was 2.7 ± 0.4 cm. Previously, stride length based on an IMU in a shoe could be estimated with a mean absolute error of 7.6 cm [31]. DFOD has a mean absolute displacement error of 1.4 ± 0.2 cm in the forward direction. Hence, displacement errors of DFOD for the tibia sensor are smaller than those reported by literature for the foot segment in running.

DFOD estimates the displacement of a sensor on the lower leg. However, the displacement of each point on the tibia can be estimated based on the orientation of the sensor and the distance from the sensor to the point of interest. When the distance from the sensor to the ankle joint is known, the forward (step length) and upward (step height) displacement of the ankle can be estimated. Running velocity can then be obtained with the step length and cycle time. Hence, DFOD provides insight into the 3D trajectory of the lower leg during running and can be used to estimate step length, step height, and running velocity based on a single IMU on the lower leg.

### 4.3. Algorithm Characteristics

The assumptions that treadmill running is a quasi-cyclical and quasi-2D movement seem to hold based on the standard deviation of the cycle times (0.8–1.9% of the cycle time) and the explained variance of the first principal component for the angular velocity in $\Psi^{s}$(84.6–95.8%). The explained variance shows that DFOD is capable of accurately estimating orientation and displacement even when 15% of the angular velocity in $\Psi^{s}$ occurs outside the 2D plane of a movement.

The effect of computing the functional mediolateral Equation (4a) and vertical Equation (4b) axes based on different numbers of gait cycles was found to be very small. The 1D orientation and displacement errors differed only 0.2° and 1.9 cm between the best- and worst-performing combination of the number of included gait cycles. Hence, during indoor treadmill running at a constant velocity, the number of gait cycles for the vertical and mediolateral axes has a limited influence on the results of DFOD.

However, the goal is to apply DFOD in less controlled environments such as outdoor running. Outdoor running is likely to result in a less cyclical running pattern [32]. It is hypothesized that for outdoor running, a smaller number of gait cycles to compute the functional mediolateral Equation (4a) and vertical Equation (4b) axes is favored over a larger number since assumptions are less likely to be violated over shorter periods. Five gait cycles to define the vertical and mediolateral axes is expected to be a reasonable trade-off between including more data to compensate for the increased variability in outdoor running while still being able to adapt to sudden changes in the gait pattern and reduce violations of assumptions. Hence, five gait cycles for both the mediolateral and vertical axes Equation (4a,b) were used in this study as the default setting for DFOD.

To investigate the effect of sampling frequencies on DFOD, inertial data were resampled from 240 Hz to 120 Hz and 60 Hz before applying DFOD. Orientation and displacement errors drastically increased when IMU data resampled to 60 Hz were used as input for DFOD. These results indicate that DFOD provides satisfactory results for a sampling frequency of 240 Hz and 120 Hz, but not for 60 Hz.

### 4.4. Limitations

Multiple assumptions were made to create DFOD, which can be violated by running outdoors. When runners run outside, they have a less constrained gait pattern than on a treadmill [32], and can freely change their running velocity and run up or downhill, thereby violating some assumptions of DFOD. Violation of the assumption of an approximately constant cycle average velocity does not influence the mediolateral axis of $\Psi^{f}$ Equation (4a) since this axis is based on the first principal component of the angular velocity of the lower leg sensor. Additionally, this axis is not influenced by taking a turn or running in circles, since it moves with the body. However, the vertical axis of $\Psi^{f}$ Equation (4b) is influenced by a violation of the approximately constant cycle average velocity assumption. This axis is equal to the direction of the total acceleration (i.e., including gravity) over a complete number of gait cycles when the cycle average velocity is constant. When a runner accelerates or decelerates, the free acceleration will not have a zero-mean over a complete number of gait cycles and will result in an offset in the estimated vertical axis proportional to the magnitude of the acceleration or deceleration. Since five gait cycles are included to estimate both functional axes, DFOD minimizes the effect of violated assumptions and is expected to recover from a short violation of assumptions within five gait cycles.

Similarly, the assumption of a quasi-cyclical 2D movement might be violated more often in running outdoors since impact accelerations are higher when running overground compared to a treadmill [33], due to uneven terrain, stumbling, or taking a turn. DFOD will recover from short violations of the quasi-cyclical 2D movement assumption within five gait cycles. Running-induced fatigue has been shown to increase variability in the gait pattern [34]. This increased variability and less cyclicity might cause the assumptions of DFOD to be less valid in fatigued running, resulting in larger orientation and displacement errors. Since DFOD has an origin that moves with the body at the cycle average velocity, a change in elevation caused by running on a sloped surface will cause the origin of DFOD to move up or down with the body. An elevation change will be visible over time; however, the average displacement will still be zero.

This study aimed to propose and validate a new algorithm that makes use of the quasi-cyclical nature of many movements. The algorithm was tested on treadmill running data of four runners and provided satisfactory results for all runners. Hence, to test the idea of using the quasi-cyclical nature of many human movements to estimate orientation and displacement, a limited number of subjects is appropriate. However, before DFOD can be used to study running kinematics it should be validated in more runners and different conditions.

This study estimated sensor orientation and displacement during running while segment orientations might provide more insight for motion analysis. For a sensor to segment calibration, two axes that relate to both CSs are required. One of these axes is already defined in DFOD Equation (2a). The other axis could be based on the direction of the gravitational acceleration during neutral standing, in which the tibia is assumed to be vertical. However, this sensor to segment calibration does require an additional calibration procedure.

### 4.5. Future Research

In future work, DFOD should be validated in a less controlled setting, such as outdoor running, in multiple body segments, and different quasi-2D movements like cycling and skating. The influence of short violations of the assumptions of DFOD, increased variability in the gait pattern (i.e., caused by fatigue), less cyclical movements, and different speeds on estimated orientations and displacement should be assessed in (outdoor) running. Additionally, the effect of continuous drift reduction instead of a drift reduction during each gait cycle Equation (4e) could be evaluated to improve the performance of DFOD. As long as two functional axes can be defined, DFOD should be able to estimate sensor orientation and displacement. Hence, the generalized idea of DFOD could also be applied to quasi-cyclical 3D movements like swimming. For 3D movements, the validity of the functional mediolateral axis Equation (4a) based on the first principal component of the angular velocity should be assessed. This component is expected to be less pronounced in 3D versus 2D movements. Finally, a sensor to segment calibration procedure could be added to enable DFOD to calculate segment orientations instead of sensor orientations.

## 5. Conclusions

The Drift-Free Orientation and Displacement estimation method (DFOD) is proposed and validated. DFOD estimates drift-free 3D sensor orientation and displacement with a single IMU in quasi-cyclical quasi-2D plane movements with an approximately constant cycle average velocity. DFOD does not require a calibration procedure, biomechanical model, constant- or zero-velocity point, Kalman filtering, or magnetometer. Small errors in lower leg sensor orientation and displacement were found when DFOD was validated against an optical reference system in treadmill running. Hence, DFOD is a promising method for quasi-cyclical motion analysis, especially when using a minimal sensor setup.

## Figures and Tables

**Table 1:** Mean orientation differences between DFOD and OMCS for all subjects combined. RMSE refers to the root mean square difference in 3D orientation. “Difference start cycle” and “Difference end cycle” refer to the difference between DFOD and OCMS (OMCS-DFOD) at the first and last sample of the gait cycle. “∆ Maximal angle” and “∆ Minimal angle” refer to the differences in the estimated maximal and minimal orientation during each gait cycle between DFOD and OMCS (OMCS-DFOD). “∆ ROM” refers to the mean differences in the estimated range of motion during each gait cycle for DFOD and OMCS. Pearson correlation coefficients (r) are provided between brackets.

$\mathrm{Orientation}\;(\Psi^{f})$	RMSE	Mean Absolute Difference	Difference Start Cycle	Difference End Cycle	∆ Maximal Angle	∆ Minimal Angle	∆ ROM
X-axis/Forward plane	5.3 ± 1.1°	4.3 ± 0.7°	−6.1 ± 5.0°	−6.2 ± 5.1°	−4.7 ± 6.1° (r = 0.78)	3.0 ± 2.2° (r = 0.81)	−7.6 ± 4.4° (r = 0.89)
Y-axis/Sagittal plane	3.1 ± 0.4°	2.6 ± 0.3°	0.3 ± 3.7°	0.4 ± 4.0°	−0.4 ± 3.4° (r = 0.95)	−2.1 ± 1.7° (r = 0.99)	1.7 ± 3.1° (r = 0.96)
Z-axis/Transversal plane	5.0 ± 2.1°	4.5 ± 2.1°	−3.5 ± 3.4°	3.4 ± 3.7°	−3.4 ± 3.2° (r = 0.96)	2.3 ± 5.0° (r = 0.97)	−5.6 ± 2.1° (r = 0.81)

**Table 2:** Mean displacement differences between DFOD and OMCS for all subjects combined. RMSE refers to the root mean square difference in 3D sensor and cluster displacement. “Difference start cycle” and “Difference end cycle” refer to the difference between DFOD and OCMS (OMCS-DFOD) at the first and last sample of the gait cycle. “∆ Maximal displacement” and “∆ Minimal displacement” refer to the differences in the estimated maximal and minimal displacement during each gait cycle between DFOD and OMCS (OMCS-DFOD). “∆ ROM” refers to the mean differences in the estimated range of motion during each gait cycle for DFOD and OMCS. Pearson correlation coefficients (r) are provided between brackets.

$\mathrm{Displacement}\;(\Psi^{f})$	RESE	Mean Absolute Difference	Difference Start Cycle	Difference End Cycle	∆ Maximal Angle	∆ Minimal Angle	∆ ROM
X-axis/Forward	1.6 ± 0.2 cm	1.4 ± 0.2 cm	2.7 ± 0.7 cm	2.8 ± 0.6 cm	2.4 ± 0.7 cm (r = 0.72)	−1.1 ± 0.4 cm (r = 0.79)	3.5 ± 0.9 cm (r = 0.81)
Y-axis/Mediolateral	1.7 ± 0.6 cm	1.5 ± 0.5 cm	−0.3 ± 2.1 cm	−0.2 ± 2.2 cm	−0.5 ± 1.6 cm (r = 0.51)	0.6 ± 1.5 cm (r = 0.65)	−1.1 ± 3.1 cm (r = 0.59)
Z-axis/Vertical	1.6 ± 0.2 cm	1.3 ± 0.2 cm	1.9 ± 0.2 cm	2.0 ± 0.3 cm	0.0 ± 1.0 cm (r = 0.50)	−0.4 ± 0.2 cm (r = 0.82)	0.4 ± 1.1 cm (r = 0.71)

## Data Availability

The data presented in this study are openly available in 4TU.ResearchData. The data can be found here: [https://doi.org/10.4121/18394190] accessed on 13 December 2021.
